# Schizoid Fantasy: Refuge or Transitional Location?

**DOI:** 10.1007/s10615-017-0629-2

**Published:** 2017-06-02

**Authors:** Candace Orcutt

**Affiliations:** 1 313 Herrick Avenue, Teaneck, NJ 07666 USA; 2 Faculty Emeritus, The International Masterson Institute, New York, NY USA; 3 Faculty, The New Jersey Institute for Training in Psychoanalysis, 121 Cedar Lane, Teaneck, NJ USA

**Keywords:** Psychotherapy, Schizoid personality, Schizoid defense, Transitional location, Play therapy

## Abstract

The schizoid personality, a type increasingly representative of our times, lives in a detached individual world. But this retreat sometimes can offer a place of transition, serving as a creative bridge to everyday life. An extended case illustration describes a schizoid patient who was able to use a playful form of psychotherapy to move from make-believe to real relationship.

## Introduction

The ability to find some sense of security through socially detached imagination is a characteristic of the schizoid personality. It typically sets the individual apart from others, but may paradoxically offer a bridge to the larger social world. Nancy McWilliams (2011) observes that: “the most exciting capacity of the schizoid person is creativity” (p. 200), and in addition, this personal creativity may even answer the needs of society: “The arts, the theoretical sciences, and the philosophical disciplines seem to contain a high proportion of such people” (McWilliams 2006, p. 4). This intersection of the schizoid’s personal world and society is possibly no coincidence. The creative work of these apparently detached individuals may perhaps provide a round-about way of finding some form of social attachment. If this is so, could the schizoid’s ability to play with reality become a starting point for psychotherapy with the schizoid patient? This presentation will explore this hypothesis and offer a clinical illustration to support it.

## The Social Context

Popular caricature shows that the creative schizoid type has found a place—however reluctant—in human society: the eccentric and antisocial painter; the religious mystic who lives in a cave; and—the schizoid achiever of our time—the computer genius. Such figures may even advance the larger world as they seek to develop their personal space. Of course, the actual schizoid spectrum is wider than this, and varies in degrees of social withdrawal, but many representatives have gained general recognition, and even some acceptance as they have—sometimes seemingly unwillingly—served society. Consider the “enigmatic” Alan Turing, inventor of artificial intelligence, whose genius hastened the end of World War II, although his manner allowed him few friends other than his computer, Christopher (Hodges 2014).

If the time is right for it, schizoid creativity, with its ability to fantasize, may even help to articulate the forming of the collective psyche. The modern art world offers examples of this from such opposite poles as the poet, Eliot, and the rock star, Bowie, who express the schizoid inclination of our era as hollow men in a wasteland, or as aliens who have problematically fallen to earth.

But to return to the individual situation, it is curious to think how creativity, that can facilitate the constructing and living of a solitary existence, may also provide a guarded means for presenting the self to others. Eliot (1933), typically reclusive (Ezra Pound nicknamed him “Old Possum”), once reflected: “Every poet would like, I fancy, to be able to think … his own thoughts behind a tragic or a comic mask”. Bowie, more direct, explained: “Offstage I’m a robot. Onstage I achieve emotion. It’s probably why I prefer dressing up as Ziggy [his rock star alter ego] to being David” (Sandford 1997, pp. 106–107). Especially in the arts, where the fantasy world of the schizoid demonstrably intersects with society, there appears the possibility of a new sort of communication for the schizoid. In the arts especially, creativity offers a compromise expression that can ease what has come to be accepted as the schizoid predicament of being neither quite here nor there.

## Supportive Theory

It is somewhat puzzling to note the relative scarcity of psychotherapeutic writings on the schizoid personality. Perhaps, as McWilliams (2011) notes, “… much commentary on schizoid conditions is buried in writings on schizophrenia” (p. 213). It would probably be useful for the literature to make a clearer distinction between these two importantly different psychic conditions. But fortunately, the British object relationists have left us insight into the subject that is original, detailed and profound. [Seinfeld (1991) also gives substantial attention to the object-relations approach to the schizoid state.].

Guntrip (1969), familiar with the schizoid predicament because he lived it, termed it the “’In and Out’ Programme,” and described it as *“The chronic dilemma in which the schizoid individual is placed, namely that he can neither be in a relationship with another person nor out of it…”*(p. 36, italics his).

Extensively presenting his own findings, and in basic agreement with his colleagues Fairbairn and Winnicott, Guntrip (1969) understands that the schizoid, as all human beings, longs for human relationship, but withdraws out of anxiety learned long ago because of *“the inability of the weak infantile ego to stand its ground and cope with outer reality* in the absence of adequate maternal support” (p. 102). Withdrawal, however, is equally intolerable, and so reaching out is repeated, fearfully retracted, and so on. The use of fantasy is necessitated by the unmet need for others: “The more people cut themselves off from human relations in the outer world, the more they are driven back on emotionally charged fantasied object-relations in their inner world. The real loss of all objects would be equivalent to psychic death” (Guntrip 1969, p. 20).

However, a life too detached from others again activates the need to reach out. Guntrip (1969) continues: “[the] ‘alternating in and out policy’ makes life extremely difficult, so that we find that *a marked schizoid tendency is to effect a compromise in a half-way-house position, neither in or out”* (p. 61; italics his).

Extending this thinking, might it not be hypothesized that the longing for relationship together with a developed capacity for fantasizing might combine to provide more than an escape into the inner world? That there might be a more constructive aspect to this defense that is able to establish a tentative “compromise” communication through creative pursuits? And perhaps—given its full potential and within a therapeutic setting—this use of fantasy to find a connection with others can go beyond compromise to create a “safe-enough” transition to real relationship?

Here we turn to Winnicott for his unique and deep insight into the schizoid state. Winnicott’s understanding was based on extensive pediatric work, experience with “severely regressed patients,” and soul-searching of his own personal condition (Rodman 2003, pp. 289–291). Like Freud, he believed that psychopathology was a manifestation of something gone awry in healthy human potential—that the abnormal could be comprehended through better understanding of the normal. Thus, Winnicott (1960) believed that the schizoid state—with its problems in relationship—has its origin in the early infant’s first attempt to relate itself to the “maternal environment.” He describes this as the “gesture” that, as a “spontaneous impulse” from the True Self, needs to be met by a receptive, non-intrusive mother, thus encouraging the infant’s further exploration into “Not-Me” space (pp. 144–146). [This explication of the infant’s first distinction between inner and outer “worlds,” self and other, is reinforced by Mahler et al.’s (1975) pediatric observations of what she calls the “differentiation subphase” of early human growth (pp. 52–64)].

When the infant’s spontaneous gesture is not well enough received, distinction between the subjective reality of the self and objective outer reality is unsure. The schizophrenic significantly fails to master this crucial developmental task. The schizoid personality, however, has been able to make a workable differentiation, but not securely. Depending on the degree to which the early experience has been wanting, the schizoid personality will regard the separateness of inside and out, self and other as a risky business. Winnicott, it should be noted, although he tends toward an inclusive description of the origin of schizoid states, clearly distinguishes between schizophrenia and characterological forms of the schizoid condition.

Good enough reception of the spontaneous gesture then makes possible the infant’s creation of “transitional space,” with its occupant, the “transitional object” (comfortably represented by the Teddy Bear). The transitional object is just that: an introduction to the real object. The transitional space it occupies is a tentative area where the first interactions, for instance via Teddy, can be experimented with on the infant’s terms. Transitional space allows the creative treating of “reality” any chosen whichway—a practice area that strengthens the infant’s confidence to explore the environment and the growing perception of the other. We carry the transitional space with us in our mature psyche, as well, says Winnicott (1951), where it “⋯shall exist as a resting-place for the individual engaged in the perpetual human task of keeping inner and outer reality separate yet inter-related” (p. 230). In the transitional space in our minds, we maintain the ability to toss about trial concepts of reality, conforming them to our notions, and illusively reassuring ourselves that external reality will provide the same responsiveness. This touches upon Winnicott’s (1952) profound concept of play. Play, he believes, defines the activity in the transitional space. It reinforces the infant’s expectation that the spontaneous gesture will be met by the receptive environment, just as Teddy’s reliable cooperation complements the infant’s behavior. We need this ability to practice and feel reassured if we are to face bigger and less cooperative challenges, and the infant’s play carries through into such adult endeavors as art, science and religion, which give us the courage to feel we can face larger realities (p. 224). Winnicott (1967) sums up the importance of transitional space, saying: “I suggest that the time has come for psycho-analytic theory to pay tribute to this *third area*, that of cultural experience which is a derivative of play” (p. 372).

It would seem that the schizoid personality, still engaged in the uncompleted task of making a good enough separation between inner and outer reality, takes up refuge in transitional space, rather than using it to move forward. There, the schizoid can create a personal world of intellectual invention or artistic fantasy, where he is free to play with the possibilities of a self-invented reality rather than test out his greater surroundings. How can therapy help to restore the individual’s true use of transitional space: from defensive refuge to a bridge to the real world?

## A Clinical Approach

First of all, what would the therapeutic situation be like? Ralph Klein (1995) has contributed substantially to the Masterson Approach to treatment of the schizoid “Self-in-Exile” (pp. 1–142). Describing the clinical work, he emphasizes that “the key word for such patients is ‘safety.’” He continues: “⋯such patients require a therapeutic alliance in which they are truly free to establish their own distance and regulate the pace of their feelings, thinking, and acting” (p. 123). McWilliams (2006), referring to an unpublished paper by Gordon, adds that “⋯ most of what is transformative to schizoid individuals involves the experience of *elaborating the self in the presence of an accepting, nonintrusive, but powerfully responsive other”* (p. 17).

Both these accounts suggest that therapy with the schizoid begins where the early developmental situation faltered. The therapist must be receptive to the patient’s self-expression in a way (to use Winnicott’s words) that is “holding” without “impinging.” Other essential words used by Klein and McWilliams are “safety” and “experience.” The patient must feel safe from interpersonal pressure, and understand this sense of safety comes not so much from words as from the experience of the therapist’s concern, full attention, and interest.

How does this go in session? The therapist begins with showing interest in what is of interest to the patient. This may be as pertinent as the patient’s intellectualized ideas about the presenting problem, or may diverge to topics of special interest to the patient such as books or computer games. This is to establish a mutual safe space with the patient, where verbal transaction can take place in an uncommitted way. This uses what Ralph Klein (1995) has called the schizoid patient’s ability to form “relationships by proxy” and so defensively “act against the risks involved in connecting to, and sharing with the therapist” (p. 90). This ability can allow the patient to test out a harmless reciprocity in the sessions. Over time, the experience of this interplay may prove to feel safe enough so that the patient may begin to further test the possibility of a closer exchange. If all goes well, interchange becomes therapy as protective strategy transforms into relationship. [The extensive examples of therapeutic dialogue with a schizoid patient that I describe in my own work (Orcutt 2012) may offer a useful guide to this technical approach (pp. 126–147).].

Those who practice play therapy will see a similar process at work here. Therapy with the schizoid individual relies on engaging the creative playfulness in the patient’s inner space in order to bridge the anxiety of exploring outer reality.

And now I would like to introduce you to a patient of mine who transformed a major city hospital into a transitional space for personal change.

## A Case Example

This is about a playful young patient I have always referred to as “the boy on the bicycle.”

Although the experience dates to my early career, it remains vividly with me. It represents a time I had to rely on intuition more than expertise, although perhaps that was a good thing. At the time, even my training needed constantly to catch up with itself, since this was a case of personality disorder, and the importance of preoedipal states was barely taking hold, while the concept of personality disorder was just forming. In any event, this case provided part of my initiation as clinical social worker on the staff of a large psychiatric hospital in a major city.

The patient, an 18-year-old male, was perceptively diagnosed by the resident conducting the intake as having “a schizoid reaction to a schizophrenic mother,” and was referred to the psychiatric outpatient facility for treatment.

My first view of mother and son walking toward me stays distinctly in my mind. The mother—tiny, anxious, and looking older than her age—was accompanied by her would-be-adult child, who was 6 feet tall, somewhat heavy, and seemed somehow larger than life. When I interviewed them together, the mother did the talking while the son appeared distracted. The problem focused essentially on the mother’s feeling overwhelmed by her son’s energy, unpredictable activity, and general unmanageability.

It seemed to me that the issue was less that of the mother’s anxiety than the son’s need for independence. And so, after a supportive session with the mother, I arranged to see the son individually. The mother, a functioning chronic schizophrenic, seemed relieved by my reassurance that her uncertain maternal instinct had rightly sent her to seek assistance in guiding her 18-year-old toward growing up, perhaps even to being on his own. I anticipated my meeting with the son with a good deal less confidence than I communicated to the mother.

The patient prefaced his first session by riding his bicycle down the Hospital hallway. He was confronted by a clerk, who protested: “You can’t ride your bicycle inside the Clinic!” The patient then cheerfully replied: “Lady! You’re hallucinating! This isn’t a bicycle, this is a horse!”

The patient had set the stage, and it was my job to make it a setting for psychotherapy.

First, there was the matter of the horse. Making it clear that a horse must be hitched outside, I went with the patient while he cooperatively chained his bicycle to the iron railing which was fortunately located at the side entrance to the Clinic.

His new experience seemed to engage the patient’s attention, and he soon informed the staff and myself that his name was “Barnabas.” This was not his actual (rather stodgy) name, but the name he took from Barnabas Collins, the reluctant vampire in the popular television series, *Dark Shadows*.

And here we coincided. In my own teenage years, I myself escaped into a world of classic horror films such as *Dracula*. To follow this inclination, I played hooky to the extent that I still wonder how I managed to graduate high school. It was a single-minded necessity for me—impulsively followed, but with such care that I never got caught. Looking back, I imagine that the way these films dramatically presented the ambiguity of good and evil, even the elusive nature of reality, helped me find my way through some complicated feelings. Unquestionably, my personal experience helped me to create a bond with Barnabas. I could speak his language.

As the sessions began, we enthusiastically discussed *Dark Shadows*, and the dilemma of unwilling vampires such as Barnabas Collins, who was struggling to free himself from a spell. At the same time, the patient continued to entertain the staff by appearing in a cape and top hat (the bicycle remained hitched outside).

Fairly soon, the fantasy play expanded to include me. We became Steed and Peel, the sophisticated crimestoppers on the original television series of *The Avengers*. He pictured himself as the dapper John Steed, with cane and bowler hat, while I was flatteringly cast as Steed’s companion, the jump-suited karate expert, Emma Peel. Now fantasy allowed us to play with relationship in metaphor.

I should be clear that these sessions provided a context for consciously constructed fantasy. The patient had no thought disorder, and knew the difference between fantasy and reality, although he approached reality with caution and in camouflage.

Then the scope of the sessions expanded further, as Barnabas instigated an activity we came to call “scavenging” (we never verbally defined this, but it is interesting to note that the word suggests the reclaiming of something that has been considered worthless). Barnabas led me on an extensive exploration of apparently forgotten regions of the Hospital. It was evident he was showing me “secret” rooms he had previously discovered, as he appeared to be confidently following a mental map. We ventured through one neglected room after another—dusty, cobwebbed places filled with stacks of papers, broken furniture, dingy test tubes and alembics. He scavenged one or two mad-scientist-looking beakers for his home use, while my white hospital coat officialized the activities.

Barnabas, with my cooperation, had transformed the Hospital into an extensive playroom.

And, taking the desired direction of play therapy, our finding of hidden inanimate objects progressed to a Clinic-wide game of interpersonal hide-and-seek.

On regular occasions, the Hospital Department of Psychiatry conducted Professor’s Rounds in a large auditorium. Over a 100 white-coated psychotherapists were there—psychiatrists, psychologists, social workers—to hear a distinguished speaker and discuss cases. I was seated toward the back, when I noticed a number of the staff turning to look at me. I also noticed that they seemed amused. My own perplexed gaze traveled toward the auditorium door, where I saw Barnabas standing and smiling at me.

I hurried to the entrance and gestured him outside. I then subjected him to a solemn boundary-setting lecture. He took it in and looked pleased.

On a later occasion, during a smaller social work meeting, Barnabas appeared at the door, singled me out, and called “Hiya, Candy!” My boundary-setting lecture to him was repeated, to his repeated satisfaction, while I noted to myself that Barnabas had somehow learned my nickname from the staff. I also noted that he had addressed me by my real name.

This increased my growing awareness that Barnabas had engaged the staff of the Hospital, as well as their setting, in his play. The staff knew him, knew I was his therapist, was aware of who knew what about our activities, and was in good-natured collusion. Barnabas had somehow engaged them in transforming an impersonal psychiatric setting into his own supportive play world.

Looking back, now even more than then, I realize that the therapeutic work had followed a definite progression: from a solitary imaginary self to an imaginary law-enforcing couple; then next, from actual companions interacting in an inanimate location to two people engaged in a kind of rapprochement in a social surrounding. Barnabas had set the scene, I had accepted and helped shape it, yet basically all we had done was make room for the orderly nature of the self to grow.

There was consistently a more conscious part to the process, as well. Each session ended in my office, where Barnabas increasingly examined his real-life situation and made a plan for his immediate future.

He had befriended his landlady, who owned a house outside the City. Barnabas was adapting well to weekend visits, and was finding himself useful around the property. In time, he and the landlady arranged for him to work as a live-in caretaker there.

Our sessions were coming to a close, but a couple of recollections remain vividly with me.

One day Barnabas brought me a jar with a bug in it. It was some sort of fragile flying creature, all legs, that hit itself futilely against the glass container. “Isn’t that the biggest damn mosquito you ever saw?” he asked. He then disappeared on some momentary task (a tendency of his) while I stared at the bug. Finally, uncertainly and guiltily, I removed the cover and let the bug fly out the window. When Barnabas returned, I apologized, and said “I just couldn’t let it stay caught in that jar.” Barnabas did not object, but remained very quiet with me for a minute.

I also recall our parting. As usual, I went with him to the side entrance of the Clinic, where he unchained his bicycle. We talked awhile, and then I started to say goodbye. He stopped me, saying “I’m not ready yet.” We stood together briefly in silence. Then he wished me well, jumped on his bicycle, and rode off.

## Conclusion

The “boy on the bicycle” taught me, as a beginning clinician, the therapeutic importance of joining with the schizoid patient in the individual’s area of personal creativity. This psychic region, so eloquently described by Winnicott as “transitional space,” exists in us all as a “third” not-me, not-other mental “location.” Here, we can shape our perception of outer reality in trial constructions under our imaginative control. This “illusory” activity eventually facilitates our participation in the far less manageable outer environment.

Beginning with Fairbairn (1946), the British object relationists revolutionized psychoanalysis by asserting that relationship with the other is the fundamental need of the self (pp. 30–37). As Winnicott says, the spontaneous gesture of the early infant initiates this process of object-seeking. However, the mother’s capacity to hold this gesture receptively is critical for the evolution of the process, through the infant’s interplay with the transitional object to the adult’s capacity for relationship with others. In the schizoid individual, the early reciprocal situation has been sufficient to allow the infant’s creating of transitional space, but not good enough to carry through to a confident capacity for relationship. Thus, to a greater or lesser extent—depending on the degree of accomplishment within the early situation—the schizoid adult retains the ability to use transitional space as a creative refuge, but is lacking in assurance to use it effectively for its transformative purpose.

The function of psychotherapy with the schizoid patient, consequently, first requires the therapist to meet with the patient in the safely defended area of transitional space (i.e.: the patient’s interests, endeavors, ideas, etc.). In this area, which is essentially under the patient’s psychic control, therapist and patient engage in a trial interpersonal experience. The intent is that this interaction will prove reassuring enough to encourage the patient toward real relationship through development of the transitional ability that has always been potential in the patient’s self.

Play, as Winnicott describes, is the essential activity within transitional space. Through creative fantasy, play first forms a safe base for interpersonal exploration, and then provides a pleasurable means for entering into relationship itself. Winnicott sees play as vital not only to the individual’s growth into a healthy social being, but also to the development of society itself (which is the greater collective task).

The boy on the bicycle introduced these concepts to me through our mutual experience which was significant for us both. He gained an increased sense of self and self-reliance through building relationship with another. I, in turn, was witness to the persistence of the spontaneous gesture of the self toward the other, and the playfulness that is inherently ready to enable this process toward relationship with creative energy and even joy.
